# High-Precision Optical Angle Detection Method for Two-Dimensional MEMS Mirrors

**DOI:** 10.3390/mi16121346

**Published:** 2025-11-28

**Authors:** Longqi Ran, Yan Wang, Zhongrui Ma, Ting Li, Jiangbo He, Jiahao Wu, Wu Zhou

**Affiliations:** 1 School of Mechanical and Electrical Engineering, University of Electronic Science and Technology of China, Chengdu 611731, China; 2 School of Mechanical Engineering, Xihua University, Chengdu 610039, China; 3 Huawei Technologies Co., Ltd., Shenzhen 518129, China

**Keywords:** MEMS, vibrating mirror, photoelectric sensor, LiDAR

## Abstract

As a core component of MEMS LiDAR, the 2D MEMS mirror, with high-precision optical angle detection, is a key technology for radar scanning and imaging. Existing piezoresistive detection schemes of mirrors suffer from high fabrication complexity, high temperature sensitivity, and a limited accuracy of only 0.08°, failing to meet the requirements for vehicular and airborne scanning applications. This study focuses on a two-dimensional electromagnetic MEMS mirror. Based on the reflection principles of geometric optics, angle detection schemes with photodiode (PD) arrays are analyzed. A novel four-quadrant optical measurement sensor featuring a 16-PD array is proposed. This design replaces conventional large-area PDs with a compact PD array, effectively mitigating nonlinearity and low accuracy issues caused by oversized PD trenches and edge dimensions. High-precision detection of the mirror’s deflection angle is achieved by measuring the current variations induced by the reflected spot position on the PDs in each quadrant. The experimental results demonstrate that the 16-PD array optical angle sensor achieves an accuracy between 0.03° and 0.036° over a detection range of ±8°.

## 1. Introduction

Two-dimensional micro-electro-mechanical system (MEMS) mirrors, fabricated via semiconductor processes, primarily consist of a micro-mirror, torsion beams, support structures, and micro-actuators. They exhibit many advantages such as their compact size, low cost, high level of integration, and high reliability [1,2]. They serve not only as primary optical scanning components in automotive LiDAR [3,4] but also find applications in optical communication and optical switching [5,6]. MEMS mirrors control light paths to specific reflections or scanning angles by driving mirror rotation [7], thus a precise angle detection is crucial for scanning sensing [8,9]. Current angle sensors for 2D MEMS mirrors include contact and non-contact ones. Contact sensors are primarily piezoresistive [10], while non-contact types include capacitive and optical sensors [11,12]. Piezoresistive and capacitive sensors exhibit a large temperature drift, low detection accuracy, measurement hysteresis, and strong biaxial coupling, rendering them unsuitable for high-reliability closed-loop control applications like LiDAR that require real-time angle feedback [13]. Optical sensors, known for their high precision and stability, represent one of the optimal angle detection solutions for 2D MEMS mirrors in automotive LiDAR. In 2007 and 2009, Ishikawa et al. proposed optical sensors comprising a Vertical-Cavity Surface-Emitting Laser (VCSEL) and photodiodes (PDs), achieving single-axis rotation detection within ±2.5° [14] and dual-axis rotation within ±1.5° [15], respectively. In 2018, Cheng et al. designed an integrated four-quadrant optical sensor for scanning micromirror angle measurement [16], demonstrating a linear response from −5° to 5°. In 2020, Zhan et al. utilized a VCSEL and nine PDs to construct an optical displacement and angle sensor for piezoelectric vibrating stages [17]. Difference-sum ratio calculations of corresponding PD outputs yielded relatively linear results, achieving a displacement range of 500 µm with 150 nm accuracy, and an angle range of ±2° with 0.1° accuracy. However, existing optical detection sensors are often bulky, offer relatively low precision, have limited angular ranges, and exhibit poor linearity, preventing their direct application in MEMS mirror systems.

A planar four-quadrant detection scheme employing 16 PDs is proposed to address automotive application demands. This approach overcomes issues associated with conventional four-PD layouts, such as a small photosensitive area, low sensitivity, and poor linearity, thereby enhancing mirror rotation angle measurement accuracy at the microscale. The design facilitates integration into micro-mirror systems, offering a feasible solution for precise control of 2D mirror scanning.

## 2. Design of Optical Detection

**Figure 1:**
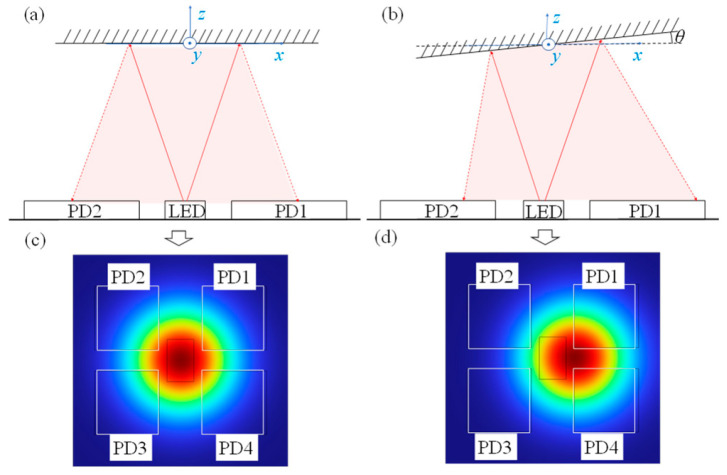
Sensor detection schematic.  (**a**) The path of the reflected light when the mirror has no rotation; (**b**) The path of the reflected light when the mirror rotates by an angle of *θ* along the y-axis; (**c**) Position of the reflected light spot when the mirror has no rotation; (**d**) Position of the reflected light spot when the mirror rotates by an angle of *θ* along the y-axis.

Conventional optical detection sensors typically utilized a four-quadrant, four-PD layout as shown in Figure 1 [9,18]. The four PDs are mounted in the four quadrants of a plane parallel to the micro-mirror surface, with the laser source positioned at the coordinate origin, emitting Gaussian beam profiles perpendicular to the *z*-axis. The light intensity distribution is expressed as:

(1) $$I=\frac{2P_{0}}{\pi \omega^{2}}\exp\left(-2\frac{r^{2}}{\omega^{2}}\right)$$

where $P_{0}$ is the total light intensity from the laser, ω is the beam radius, and r is the radial distance from the beam center. Assuming the light intensities received by PD1 to PD4 are $I_{1}$, $I_{2}$, $I_{3}$, and $I_{4}$, respectively, when the measured surface tilts, its deflection angles along the two sensitive axes correspond to variations in the received light intensities of the four PDs.

**Figure 2:**
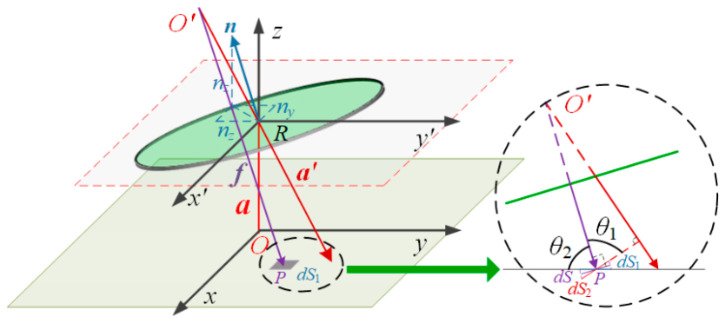
Sensing principle of the sensor.

The two-dimensional angle conversion detection of the optical sensor is described in the x-y-z coordinate system (Figure 2). The light source is located at the origin O(0,0,0), with a beam divergence half-angle of $\theta_{\mathrm{beam}}$. The mirror surface is positioned at a distance h above the light source, capable of rotating about the *x*-axis and *y*-axis by angles $\theta_{x}$ and $\theta_{y}$, respectively. The mirror surface normal vector is represented as:

(2) $$n={\left(C_{y}\;\;C_{x}\right)}^{T}\left[\begin{array}{c} 0\\ 0\\ 1 \end{array}\right]=\left[\begin{array}{c} \sin\theta_{y}\\ -\cos\theta_{y}\sin\theta_{x}\\ \cos\theta_{y}\cos\theta_{x} \end{array}\right]=\left[\begin{array}{c} n_{x}\\ n_{y}\\ n_{z} \end{array}\right],$$

where $C_{x}$ and $C_{y}$ are the mirror rotation matrices, expressed as:

(3) $$C_{x}=\left[\begin{array}{ccc} 1 & 0 & 0\\ 0 & \cos\theta_{x} & \sin\theta_{x}\\ 0 & -\sin\theta_{x} & \cos\theta_{x} \end{array}\right],\;\;C_{y}=\left[\begin{array}{ccc} \cos\theta_{y} & 0 & -\sin\theta_{y}\\ 0 & 1 & 0\\ \sin\theta_{y} & 0 & \cos\theta_{y} \end{array}\right],$$

The center of the light spot on the *Oxy* plane can be considered as the projection of the light source’s mirror image point $O^{\prime }$ along the vector $a^{\prime }$ onto the *Oxy* plane. According to geometric optics, the coordinates of the mirror image point are calculated as:

(4) $$O^{\prime }=2\left(a\cdot n\right)n=\left[\begin{array}{c} 2hn_{x}n_{z}\\ 2hn_{y}n_{z}\\ 2hn_{z}n_{z} \end{array}\right],$$

The reflected beam vector is thus:

(5) $$a^{\prime }=R-O^{\prime }=\left[\begin{array}{c} -2hn_{x}n_{z}\\ -2hn_{y}n_{z}\\ h-2hn_{z}n_{z} \end{array}\right],$$

The PD is located at a height $h_{p}$. Since the reflected beam does not illuminate the PD vertically (Figure 2), the received intensity must be calculated by integrating the intensity over infinitesimal area elements, *dS*. The projection distance l of vector f onto vector $a^{\prime }$ is given by the geometric relation:

(6) $$l=f\cdot \frac{a^{\prime }}{\left|a^{\prime }\right|}=\frac{\left|2n_{z}n_{x}\left(2hn_{z}n_{x}-x\right)+2n_{z}n_{y}\left(2hn_{z}n_{y}-y\right)+\left(1-2n_{z}n_{z}\right)\left(2hn_{z}n_{z}-h_{p}\right)\right|}{\sqrt{{\left(2n_{z}n_{x}\right)}^{2}+{\left(2n_{z}n_{y}\right)}^{2}+{\left(1-2n_{z}n_{z}\right)}^{2}}},$$

The perpendicular distance r from the infinitesimal area element dS to vector $a^{\prime }$ is:

(7) $$r=\sqrt{{\left|f\right|}^{2}-{\left|f\cdot a^{\prime }\right|}^{2}}$$

The beam radius at the projection distance l, considering the source divergence half-angle, is:

(8) $$\omega =l\tan\theta_{beam}$$

At point P, the light intensity of each infinitesimal area satisfies:

(9) $$\begin{array}{l} IdS=I_{1}dS_{1}=I_{2}dS_{2}\\ I=\frac{I_{1}dS_{1}}{dS}=\frac{I_{1}}{\sin\theta_{1}}\\ I=\frac{I_{2}dS_{2}}{dS}=\frac{I_{2}}{\sin\theta_{2}} \end{array}$$

The infinitesimal element *dS*_2_ is perpendicular to the reflected light, and its light intensity distribution satisfies Equation (1). Therefore, substituting Equations (7) and (8) into Equation (1) provides the light intensity distribution of the infinitesimal element *dS*_2_. Furthermore, according to Equation (9), the light intensity distribution of PD can be obtained as:

(10) $$P=\int I_{1}dS_{1}=\int I\frac{\sin\theta_{1}}{\sin\theta_{2}}dS_{2},$$

## 3. Detected Angle Calculation

**Figure 3:**
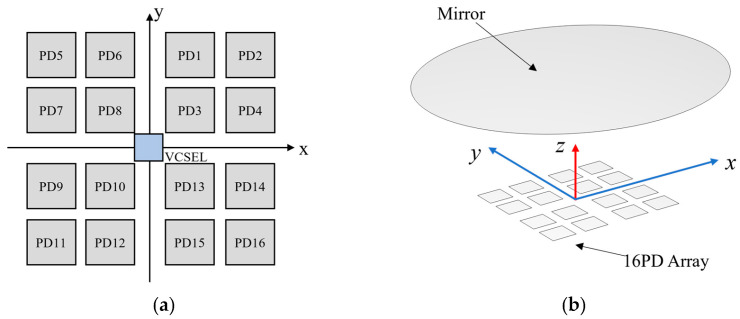
Optical detection theme: (**a**) layout of PD array; (**b**) simulation model.

To establish a more linear and monotonic relationship between the angle change and the received light intensity change, the difference-sum ratio method is employed, converting the received light intensities from the four PDs into variation signals. To address the nonlinearity and low accuracy inherent in large four-quadrant PD layouts, this paper adopts an array of small-area PDs to replace a single large-area PD per quadrant, as illustrated in Figure 3a. The sum of the received light intensities from the four small PDs in a quadrant can be treated as a single unit for calculating the current difference-sum ratio. This method effectively mitigates the accuracy degradation caused by the large PD trench and edge dimensions, enabling precise angle detection. The angle variation can be calculated from the current difference-sum ratio given by Equation (10).

(11) $$\left\{\begin{array}{l} R_{x}=\frac{\left(I_{A}+I_{B})-(I_{C}+I_{D}\right)}{\left(I_{A}+I_{B})+(I_{C}+I_{D}\right)}\\ R_{y}=\frac{\left(I_{A}+I_{D})-(I_{B}+I_{C}\right)}{\left(I_{A}+I_{D})+(I_{B}+I_{C}\right)} \end{array}\right.,$$

where *R_x_* and *R_y_* represent the difference-sum ratios of currents corresponding to the rotation of the mirror about the *x*-axis and *y*-axis, respectively. *R_x_* = 0 indicates no rotation about the *x*-axis, while *R_y_* = 0 signifies no rotation about the *y*-axis. $I_{A}=I_{1}+I_{2}+I_{3}+I_{4}$, $I_{B}=I_{5}+I_{6}+I_{7}+I_{8}$, $I_{C}=I_{9}+I_{10}+I_{11}+I_{12}$, $I_{D}=I_{13}+I_{14}+I_{15}+I_{16}$.

## 4. Simulation Analysis

Compared to the single-axis difference-sum ratio of 1D mirrors [17], the 2D MEMS mirror involves two sensitive axes, resulting in a curved surface representing the difference-sum ratio at different mirror rotation angles. Finite element simulation was performed using the COMSOL Multiphysics 6.1 optics module. The model is shown in Figure 3b. The light source is located at the origin of the *O*-*xyz* coordinate system, emitting a laser beam along the z-direction with a divergence angle of 10° and a power of 8 mW. The beam is reflected by the mirror surface onto the PD array. Each PD has a side length of 0.25 mm, and the distance between the mirror and the PD array is 2.50 mm. The total light-receiving area per quadrant is consistent between the compared models.

**Figure 4:**
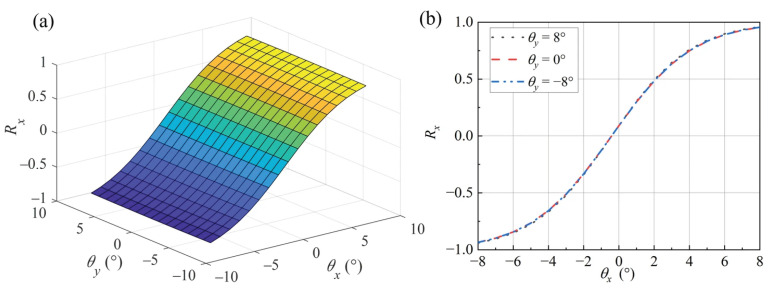
Simulation results of difference-sum ratio: (**a**) difference-sum ratio surface; (**b**) rotation about the *X*-axis.

The simulation results for the difference-sum ratio surface are shown in Figure 4a. Taking the difference-sum ratio for rotation about the *x*-axis as an example, the extracted data from the ratio surface are plotted in Figure 4b. Significant biaxial coupling and nonlinearity are observed, preventing the direct use of one-dimensional linear fitting for angle calculation. The linearity of the difference-sum ratio decreases as the rotation angle increases. The difference-sum ratio curve is slightly influenced by rotation about the non-sensitive axis (*y*-axis). For the same rotation angle about the *x*-axis, a ±8° rotation about the *y*-axis introduces a biaxial crosstalk error of approximately 0.4%.

**Figure 5:**
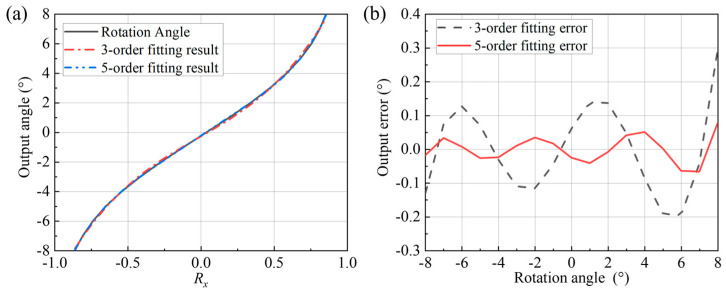
Polynomial fitting results for angle data: (**a**) fitting curves; (**b**) fitting errors.

This study employs polynomial fitting methods. Calculation results for third-order and fifth-order polynomial fits are shown in Figure 5. The fifth-order polynomial fit demonstrates significantly higher accuracy than the third-order fit, albeit with longer computation time. The specific application should determine the optimal choice based on actual requirements.

## 5. Hardware Design

**Figure 6:**
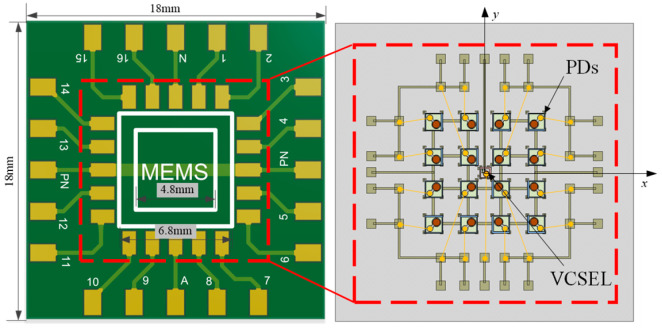
Diagram of sensor assembling and wiring.

The optical angle sensor comprises a VCSEL, PDs, and a silicon substrate. The VCSEL and PDs are bonded onto the silicon substrate using conductive adhesive. The silicon substrate features alignment marks, electrodes, and conductive traces, and is itself mounted onto a designated area of a PCB with encapsulating adhesive, as illustrated in Figure 6. The PCB measures 18 mm × 18 mm. The silicon substrate is positioned within a 4.8 mm × 4.8 mm white-lined zone on the PCB, ensuring precise alignment without angular deviation during installation.

**Figure 7:**
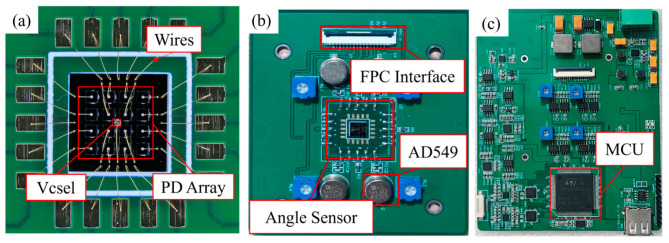
Angle detection sensor and system: (**a**) sensor chip; (**b**) sensing system; (**c**) main control chip.

The silicon substrate consists of three layers: a silicon base, a silicon dioxide (SiO_2_) insulation layer, and a metal layer. The SiO_2_ layer is formed via high-temperature oxidation, while the metal layer is deposited by magnetron sputtering. The mounting positions for the VCSEL and PDs are determined by alignment marks created through chemical etching, and the devices are attached using conductive adhesive. Bonding pads are arranged along the periphery of the silicon substrate. After the VCSEL and PDs are mounted, wire bonding is performed to connect the pads of each device to the corresponding pads on the silicon substrate. The assembly result of the PD die and VCSEL is shown in Figure 7a. The overall configuration of the sensor and its associated detection circuit system is presented in Figure 7.

**Figure 8:**
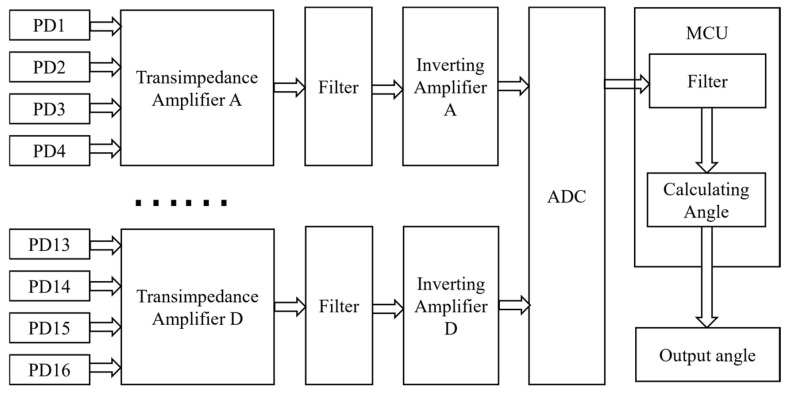
System circuit diagram.

Figure 8 illustrates the electronic architecture of the sensor module. The four PDs in each quadrant are connected in parallel. Their combined output photocurrent is first converted into a negative voltage signal by a transimpedance amplifier (TIA), then processed by a low-pass filter (LPF), and subsequently inverted to a positive voltage by an inverting amplifier. This positive voltage signal is digitized by an ADC (analog-to-digital converter). A microcontroller unit (MCU) acquires the digital data, implements digital filtering and angle calculation algorithms internally, and finally transmits the results to a host computer (PC).

## 6. Experimental Verification

**Figure 9:**
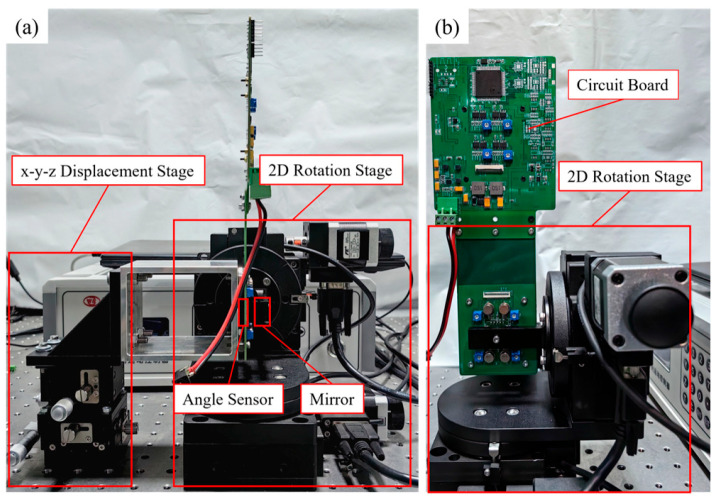
Sensor test platform. (**a**) Front view; (**b**) Right view.

The experimental setup for testing the sensor’s detection accuracy is shown in Figure 9. The test bench was fixed on an optical platform. The detection circuit board equipped with the optical sensor was mounted on a three-axis translation stage via a mechanical fixture, allowing alignment with the rotation center of the dual-axis rotation stage by adjusting the stage’s position in three dimensions. To verify the sensor’s angle measurement performance, a square silver-coated silicon wafer was used to simulate the MEMS mirror, reflecting the laser beam emitted from the sensor’s VCSEL. A dual-axis rotation stage drove the silicon reflector to rotate, mimicking the mirror’s operational state. The reflector fixture was mounted at the rotation center of the dual-axis stage. The stage used was a high-precision electric rotary stage (Model: WN02RA100S-180) with a grating encoder resolution greater than 0.005°, biaxial perpendicularity deviation less than 0.05°, and coincidence error less than 0.2 mm, meeting the precision requirements for mirror operation. The dual-axis stage was connected to a host computer to receive rotation commands and transmit real-time angle information from the encoder. The detection circuit board was connected to the host computer via an I/O-to-USB cable, sending the current voltage output signals from the sensor. During the experiments, the VCSEL at the center of the optical angle sensor emitted a beam towards the reflector. The reflected beam, carrying information about the reflector’s rotation angles, formed a Gaussian spot on the sensor surface. The photosensitive surfaces of the PDs in the four quadrants converted the received photons into currents, which were then amplified and converted into voltage outputs.

**Figure 10:**
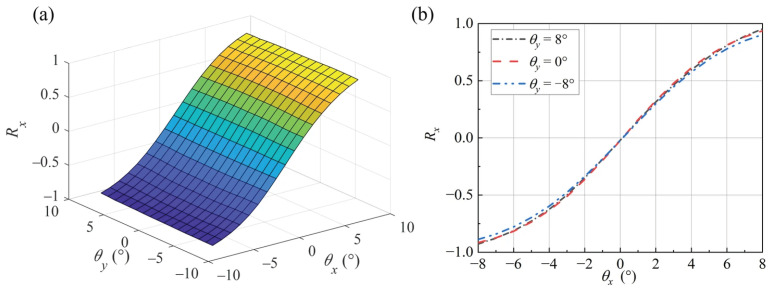
Experiment results of difference-sum ratio: (**a**) difference-sum ratio surface; (**b**) rotation about the *X*-axis.

During the experiment, the mirror deflection angles were scanned in a biaxial stepping manner within a predetermined range. One axis of the motor was adjusted to a specified position, while the other axis was stepped through a range of angles, alternating repeatedly. The deflection range was from −8° to 8°, with a step size of 1° and a sampling time of 5 s per step. With the rotation angle about the *y*-axis fixed at −8°, the rotation angle about the *x*-axis was adjusted. The average value of the last 2 s of data at each step was taken as the final output. The rotation angles and the differences/ratios around the x-direction were shown in Figure 10. Compared with Figure 4, the experimental data exhibits greater errors when the rotation angle around the *y*-axis is larger. This is because it is difficult to perfectly align the rotation center of the rotating platform with the sensor center in practical operations.

**Figure 11:**
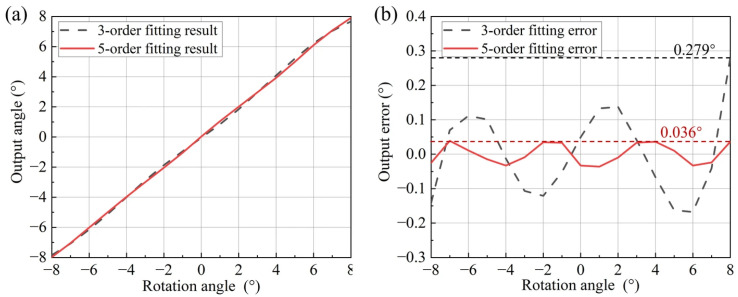
Sensor output results and polynomial fitting errors: (**a**) comparison between output angle and real angle; (**b**) comparison of output error.

After angle calculation using polynomial fitting, the maximum error was 0.279° with a root mean square (RMS) error of 0.1226° under the third-order fitting. For the fifth-order fitting, the maximum error was reduced to 0.036°, and the RMS error was 0.019°, as shown in Figure 11. The fifth-order polynomial fit yielded higher accuracy than the third-order fit. Multiple measurements with reflectors at different positions and orientations yielded the accuracy range shown in Table 1. Compared to existing piezoresistive detection methods, the proposed optical detection scheme improves accuracy by a factor of two, making MEMS mirror applications in automotive LiDAR feasible.

## 7. Conclusions

This paper addressed the low accuracy of existing piezoresistive detection methods for MEMS mirror rotation angles by designing and fabricating a non-contact optical detection sensor featuring a 16-PD four-quadrant layout. By analyzing the causes of nonlinearity in conventional 4-PD four-quadrant detection schemes, it was proposed that using an array of small PDs significantly reduces discontinuous sensing errors induced by edge effects. Furthermore, a polynomial fitting-based angle calculation algorithm was implemented to compensate for the nonlinear error in the difference-sum ratio. Finally, sensor fabrication and test platform setup were completed, achieving high-precision detection of biaxial rotation angles for the MEMS mirrors. The detection accuracy was improved from 0.08° to 0.03–0.036°, overcoming a key bottleneck in LiDAR scanning angle control and providing technical support for the application of MEMS LiDAR in automotive navigation.

## Figures and Tables

**Table 1:** Angle Calculation Results.

Performance Metric	Piezoresistive Scheme	Proposed Optical Scheme
Biaxial Coupling	~1.5%/°	<1.37%/°
Measurement Range	±7.5°	±8°
Measurement Error	>0.08° @ ±7.5°	0.03°~0.036° @ ±8°

## Data Availability

Data are available upon request via personal contact with the corresponding author via the email address zhouwu916@uestc.edu.cn.
